# The impact of productivity standards on psychotherapy

**DOI:** 10.3389/fpsyg.2023.1229628

**Published:** 2023-11-20

**Authors:** Gilbert Franco

**Affiliations:** Department of Human Services and Psychology, Beacon College, Leesburg, FL, United States

**Keywords:** psychotherapist, productivity, performance, clinician, burnout, therapy, turnover, self-efficacy

## 1 Introduction

It is a Friday afternoon, and you are a licensed mental health clinician working at a non-profit community mental health agency. You continue to stare at your computer screen and start to feel a pit in your stomach grow as you calculate your productivity for the month. Your productivity requirement is 7,200 min per month, which roughly translates to 360 min per workday. In other words, 6 h of your day must be spent in billable face-to-face time with clients. That pit in your stomach turns to dread as you realize you were 420 min short of your monthly goal, and your program manager gave you a verbal warning for not meeting your productivity goal last month.

Psychotherapists are attracted to the field of psychotherapy for various reasons. It could be that a therapist was inspired by psychotherapy that they themselves received in the past. Conversely, a therapist's motivation to enter the profession can be as simple as the desire to help other people in need. Many neophyte therapists enter the field through community mental health agencies and are introduced to a variety of performance standards to quantify their work. This quantification of a therapist's performance at work, usually measured as productivity standards, can lead to less than desired results (Franco, 2015, 2016) as therapists attempt to cope with the resulting cognitive dissonance as they juggle the quantity-vs.-quality dilemma. Hatchett and Coaston (2018) further added that productivity standards can impact a therapist's job security, which can influence the therapist's decision-making process in the quantity-vs.-quality dilemma. Additionally, Bennett et al. (2019) asserted that productivity standards negatively impact client quality of care.

## 2 Productivity standards

According to Franco (2015, 2016), productivity standards are defined as the amount of time that a therapist spends in face-to-face contact with their client. Agencies can, therefore, require their therapist employees to spend a specific number of hours per day in face-to-face contact with clients (Franco, 2016). For example, a community mental health agency can require a therapist to spend 6 to 7 h seeing clients, with the remainder of their time being spent writing notes.

A challenge with using productivity standards is that they do not take into consideration whether an employee is satisfied with their work or not (Jenaro et al., 2007). While there is research available on the relationship between employee measurement and job attitudes (e.g., Rodriguez et al., 2009a,b; Böckerman and Ilmakunnas, 2012), there is limited literature on the relationship between productivity and job satisfaction (Franco, 2015, 2016). The limited available literature has found a negative relationship between productivity and job satisfaction and a positive relationship between productivity and turnover intent (Franco, 2015, 2016).

The negative impact of productivity on therapist job outcomes has been discussed (e.g., Franco, 2015, 2016; Bennett et al., 2019). Franco (2016) concluded that managers and those designing jobs for therapists should consider redesigning jobs that address productivity standards in a manner that reduces the negative impact of productivity on therapist job attitudes. Franco (2015) finding that job self-efficacy partially mediates the relationship between productivity standards and therapist job satisfaction and turnover intent offers a clue to address this issue.

Bandura (1991) conceptualizes self-efficacy as a person's belief in their ability to perform a task and achieve their goal. Using this conceptualization of self-efficacy, one can surmise that a manager can design a therapist's job to increase their self-efficacy toward meeting productivity standards. For example, a clinical supervisor or manager can promote an open environment that encourages dialogue, cooperation, and feedback.

Implementing these strategies to increase therapist self-efficacy can lead to increased performance, but it is important to address whether the therapist will buy into the concept of productivity standards to begin with. During my career as a therapist, clinical supervisor, and clinical director, I have encountered many therapists who struggled with understanding and buying into the concept of productivity standards. A common theme that has echoed throughout my conversations with other therapists was that productivity standards did not reflect their performance as therapists. Creating an open environment for feedback can be an important step in addressing productivity standards, but in order to increase the likelihood of open and honest feedback, a manager or clinical supervisor can start by building rapport with their employees.

Rapport building involves creating a sense of trust between a manager or clinical supervisor and their supervisee through verbal and non-verbal behaviors (Curry et al., 2019). An example of verbal behavior is the use of open-ended questioning (Curry et al., 2019). Clinical supervisors can use verbal behaviors, such as open-ended questioning, to learn about a therapist's perspective on the agency's productivity standards. This, in turn, can enable supervisees to build their self-efficacy (Vandament et al., 2022). As discussed earlier, self-efficacy mediates the relationship between productivity standards and therapist job attitudes, such as turnover intent.

Non-verbal communication can also be used by clinical supervisors to build rapport and create a safe environment that promotes dialogue. Examples of non-verbal communication include eye contact, smiling, and leaning forward to show that you are expressing interest (Curry et al., 2019). In addition to verbal communication, non-verbal communication has been found to impact service provider self-efficacy (Mata et al., 2021). A manager or clinical supervisor can use non-verbal communication such as a warm smile and clear eye contact when discussing productivity standards with the therapist. With the help of non-verbal communication and open-ended questions, rapport can be built. Once rapport is built, strategies can be discussed.

Clinical supervisors can work toward preparing therapists with strategies to address meeting productivity standards. Supervisors can provide non-judgmental feedback to their therapist supervisees to process the therapist's progress in meeting productivity standards and their thoughts and feelings toward productivity standards. By giving therapists the tools to meet productivity standards and an open environment to process this abstract business concept seemingly unrelated to therapy, clinical supervisors can enable their supervisees to increase their self-efficacy.

## 3 Discussion

With all of this being said, rapport building and enhancing the self-efficacy of mental health clinicians to address the issue of productivity standards may address the issue in the short term, but perhaps it is the concept of productivity standards itself that is the main issue. Productivity standards serve as a form of performance measurement (Franco, 2016). Performance measurement is intended to increase employee performance, yet it has been found to have a paradoxical effect on mental health clinicians (Franco, 2015). In other words, instead of therapists increasing their performance to meet an agency's productivity standards, they may become burned out and suffer from low job satisfaction, which would have the opposite effect (Franco, 2016). They may even be outright fired if they do not meet their productivity quota for the month.

Hatchett and Coaston (2018) elaborated on the impact of productivity standards on a therapist's job security and stated that missed appointments from clients can lead to premature termination because it would impact a therapist's productivity. They further stated that both appointments missed by clients and therapist premature termination are common occurrences in the mental health industry (Hatchett and Coaston, 2018). Unfortunately, this results in hard and soft costs for the agencies themselves.

Hard costs include advertising and job postings for vacant therapist positions (Franco, 2015). Soft costs include lower worker productivity and morale (Franco, 2015). This can result in a downward cycle of lower morale, leading to lower productivity, which then leads to premature termination. This premature termination, which is a form of turnover, can negatively impact the client's quality of care.

Hatchett and Coaston (2018) discussed strategies such as providing financial penalties to clients who miss sessions and appointment reminders to reduce the number of missed sessions in order to increase therapist productivity. While these suggestions can serve as a band-aid and increase productivity in the short term, such approaches do not address the larger picture.

Perhaps, it is time for agencies, mental health government agencies, and policymakers to revisit the idea of productivity standards as a form of performance measurement. Government and county contracts often provide productivity standard requirements for agencies to fulfill annually as stipulations for contracts and contract renewals. As found through research, though, the result is that therapists are unsatisfied, burned out, and want to leave their jobs—all this when they are needed the most.

## 4 Conclusion

It is time to explore alternatives to productivity standards. Performance measures such as client satisfaction surveys, recidivism rates, and client outcomes measures may be more appropriate and lead to more buy-in from therapists. In my career, I have seen many therapists leave their jobs and even their professions due to stress and burnout. This is further exacerbated by a requirement that may be out of their control in many cases. A client may not show up for a session for a variety of reasons, and even one “no show” can impact a therapist's productivity.

Providing incentives to reduce no-shows or having client reminders do not address the larger picture. Performance management in the form of productivity standards is not working. Through empathy and providing non-judgmental feedback, clinical supervisors and managers can help build a therapist's self-efficacy, mitigating some of these effects, but this may not be enough. If we do not explore and implement alternative performance measures, we may be in danger of losing therapists at a time when we need them the most.

## Author contributions

The author confirms being the sole contributor of this work and has approved it for publication.

## References

[B1] Bandura A. (1991). Social cognitive theory of self-regulation. Organiz. Behav. Hum. Dec. Proc. 50, 248–287. 10.1016/0749-5978(91)90022-L

[B2] Bennett L. E. Jewell V. D. Scheirton L. McCarthy M. Muir B. C. (2019). Productivity standards and the impact on quality of care: a national survey of inpatient rehabilitation professionals. Open J. Occup. Ther. 7, 1–11. 10.15453/2168-6408.1598

[B3] Böckerman P. Ilmakunnas P. (2012). The job satisfaction-productivity nexus: A study using matched survey and register data. Ind. Labor Relat. Rev. 65, 244–262. 10.1177/001979391206500203

[B4] Curry S. M. Gravina N. E. Sleiman A. A. Richard E. (2019). The effects of engaging in rapport-building behaviors on productivity and discretionary effort. J. Organiz. Behav. Manag. 39, 213–226. 10.1080/01608061.2019.1667940

[B5] Franco G.E. (2016). Productivity standards: Do they result in less productive and satisfied therapists? Psychol. Manag. J. 19, 91–106. 10.1037/mgr0000041

[B6] Franco G. E. (2015). Productivity standards, marriage and family therapist job satisfaction, and turnover intent. Dissertations and Doctoral Studies.

[B7] Hatchett G. T. Coaston S. C. (2018). Surviving fee-for-service and productivity standards. J. Mental Health Couns. 40, 199–210. 10.17744/mehc.40.3.02

[B8] Jenaro C. Flores N. Arias B. (2007). Burnout and coping in human service practitioners. Prof. Psychol. Res. Pract. 38, 80–87. 10.1037/0735-7028.38.1.80

[B9] Mata Á. N. D. S. de Azevedo K. P. M. Braga L. P. de Medeiros G. C. B. S. de Oliveira Segundo V. H. Bezerra I. N. M.. (2021). Training in communication skills for self-efficacy of health professionals: a systematic review. Hum. Resour. Health 19, 1–9. 10.1186/s12960-021-00574-3 33676515 PMC7937280

[B10] Rodriguez H. P. von Glahn T. Elliott M. N. Rogers W. H. Safran D. (2009b). The effect of performance-based financial incentives on improving patient care experiences: a statewide evaluation. J. General Inter. Med. 24, 1281–1288. 10.1007/s11606-009-1122-6 19826881 PMC2787940

[B11] Rodriguez H. P. von Glahn T. Rogers W. H. Safran D. (2009a). Organizational and market influences on physician performance on patient experience measures. Health Serv. Res. 44, 880–901. 10.1111/j.1475-6773.2009.00960.x 19674429 PMC2699913

[B12] Vandament M. L. Duan C. Li S. (2022). Relationships among supervisee perceived supervisor cultural humility, working alliance, and supervisee self-efficacy among white supervisor and supervisee of color dyads. Train. Educ. Profess. Psychol. 16, 244. 10.1037/tep0000370

